# Biosafe Management of *Botrytis* Grey Mold of Strawberry Fruit by Novel Bioagents

**DOI:** 10.3390/plants10122737

**Published:** 2021-12-12

**Authors:** Elhagag A. Hassan, Yasser S. Mostafa, Saad Alamri, Mohamed Hashem, Nivien A. Nafady

**Affiliations:** 1 Botany Department, Faculty of Science, Assiut University, Assiut 71516, Egypt; niviennafady@aun.edu.eg; 2 Biology Department, Faculty of Science, King Khalid University, P.O. Box 10255, Abha 61321, Saudi Arabia; ysolhasa1969@hotnail.com (Y.S.M.); saralomari@kku.edu.sa (S.A.)

**Keywords:** *Botrytis cinerea*, *Bacillus safensis*, preen (uropygail) oil, strawberry, postharvest disease, innovative strategy

## Abstract

Recently, there have been urgent economic and scientific demands to decrease the use of chemical fungicides during the treatment of phytopathogens, due to their human health and environmental impacts. This study explored the biocontrol efficacy of novel and eco-friendly preen (uropygial) oil and endophytic *Bacillus safensis* in managing postharvest *Botrytis* grey mold in strawberry fruit. The preen oil (25 μL/mL) showed high antifungal activity against *B*. *cinerea* Str5 in terms of the reduction in the fungal radial growth (41.3%) and the fungal colony-forming units (28.6%) compared to the control. A new strain of *Bacillus safensis* B3 had a good potential to produce chitinase enzymes (3.69 ± 0.31 U/mL), hydrolytic lipase (10.65 ± 0.51 U/mL), and protease enzymes (13.28 ± 0.65 U/mL), which are responsible for the hydrolysis of the *B*. *cinerea* Str5 cell wall and, consequently, restrict fungal growth. The in vivo experiment on strawberry fruit showed that preen (uropygial) oil reduced the disease severity by 87.25%, while the endophytic bacteria *B*. *safensis* B3 reduced it by 86.52%. This study reports the efficiency of individually applied bioagents in the control of phytopathogenic fungi for the first time and, consequently, encourages their application as a new and innovative strategy for prospective agricultural technology and food safety.

## 1. Introduction

Strawberry (*Fragaria ananassa* Duch.; Family: Rosaceae) is an important and high-value fruit due to its unique flavor and its nutritional and health benefits [1], and is one of the most widely consumed berries in the world. However, strawberries may be exposed to mechanical injury and decay during harvesting and storage, increasing their susceptibility to various phytopathogens, including viruses, bacteria, and fungi [2,3]. *B. cinerea* is one of the most common fungal pathogens for strawberry infections, causing a destructive grey mold disease [4]. *B*. *cinerea* infects various strawberry organs in the field, including fruits during storage, resulting in poor yield quality [1,5]. Consequently, the infection of strawberries by *B*. *cinerea* causes serious economic crop loss in preharvest stages during growing seasons, harvesting time, and handling processes, as well as in postharvest stages during transportation and storage conditions [6]. It was reported that the strawberry yield loss may exceed 50% in favorable environmental conditions due to the development of *Botrytis* grey mold [7]. To date, the most efficient tactics to control the postharvest pathogen *B*. *cinerea* depend mainly on the application of fungicides [8], although there are urgent economic and scientific demands to decrease the use of chemical fungicides due to their human health and environmental impacts [9,10], as well as the development of new fungicide-resistant fungal strains [11]. As of recently, in some European countries, fungicides are fully forbidden during the postharvest stage [9]. Consequently, the discovery of new, innovative, and environmentally friendly strategies has become a vital trend to control fungal pathogens [5,12,13]. Applying different essential oils and medicinal plant extracts might be a sustainable environmental and economical solution for controlling phytopathogens due to their having little or no toxicity to humans and the environment [14]. In this context, the search for innovative, safer, and available alternatives that are derived naturally must be developed in order to decrease the postharvest fruit losses. Preen (uropygial) oil is a waxy substance extracted from birds’ feathers, and shows high efficiency in inhibiting the growth of microorganisms [15,16]. However, to date, there are no available studies concerning the application of preen oil against phytopathogens. We assume that preen (uropygial) oil may be used as a biocontrol agent to suppress phytopathogens. 

In addition, the application of microbial antagonists as biocontrol agents in managing postharvest fruit diseases is considered to be an environmentally safe and promising tactic [17]. Many antagonistic microorganisms—including bacteria, fungi, and yeasts—show high efficiency against various postharvest fruit diseases [18,19,20]. For *B. cinerea* grey mold disease management, the current study aimed to investigate the efficiency of both preen (uropygial) oil—extracted from chicken feathers—and the endophytic bacteria *Bacillus safensis*, as safe and effective alternative biocontrol agents against *B*. *cinerea*, as well as to investigate the impact of combined antagonistic bacteria and preen oil for the biocontrol of *B*. *cinerea* grey mold disease.

## 2. Results

### 2.1. Identification and Pathogenicity of the Fungal Pathogen

**Figure 1:**
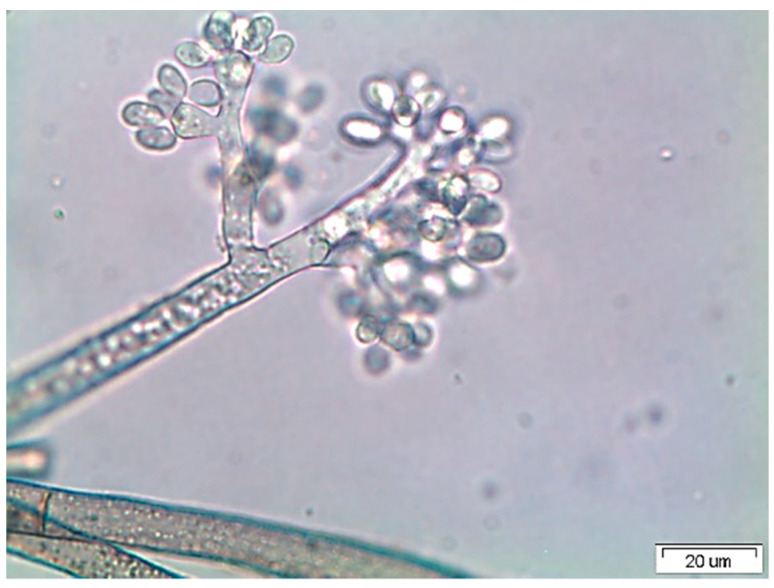
*Botrytis cinerea* Str5—the causal agent of strawberry grey mold disease.

**Figure 2:**
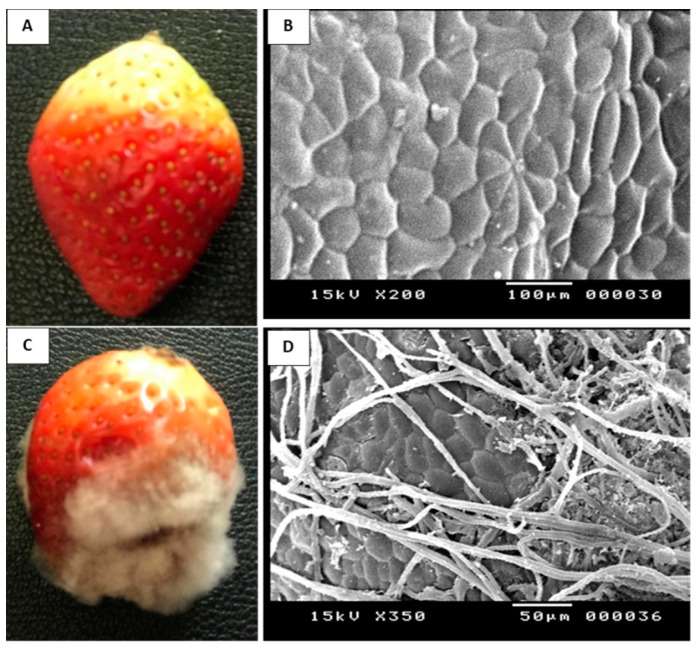
Scanning electron micrographs illustrating a comparison between healthy strawberry fruit and those infected with *B*. *cinerea* Str5. Healthy strawberry fruit show a smooth surface without fungal colonization (**A**,**B**); however, the infected fruit (**C**,**D**) show the intensive occurrence of hyphae on the fruit surface.

*Botrytis cinerea* Str5 was isolated from strawberry fruit showing grey mold symptoms. Based on the cultural and microscopic characteristics, the recovered fungus was identified as *B*. *cinerea* Str5 (Figure 1). The pathogenicity test of *B*. *cinerea* Str5 for strawberry fruit proved the severity of the isolated fungus, as well as its involvement in the development of the grey mold symptoms on the fruit. The fungus recorded a high level of colonization of the infected fruit within 5 days. The produced symptoms of the infected fruit showed a visible white-to-greyish cottony mass of the fungal mycelium, along with abundant fungal sporulation of conidiophores and fungal conidia on the rotted strawberry tissues (Figure 2). The fungal infection was also confirmed with scanning electron micrographs showing the colonization of conidiophores and fungal mycelia on the surface of the strawberry fruit (Figure 2), whereas scanning electron micrographs of uninfected strawberry fruits showed fine and smooth fruit morphology without any growing mycelia.

### 2.2. Fungicidal Effect of Preen Oil against B. cinerea Str5

**Figure 3:**
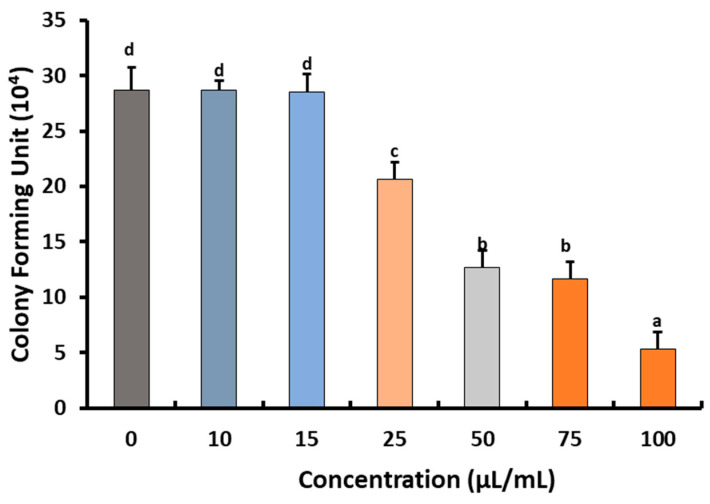
Impacts of different concentrations of preen oil on *B*. *cinerea* Str5 growth in terms of colony-forming units. Vertical bars represent the standard error (*n* = 3). Columns followed by the same letter are not significantly different at *p* < 0.05.

**Figure 4:**
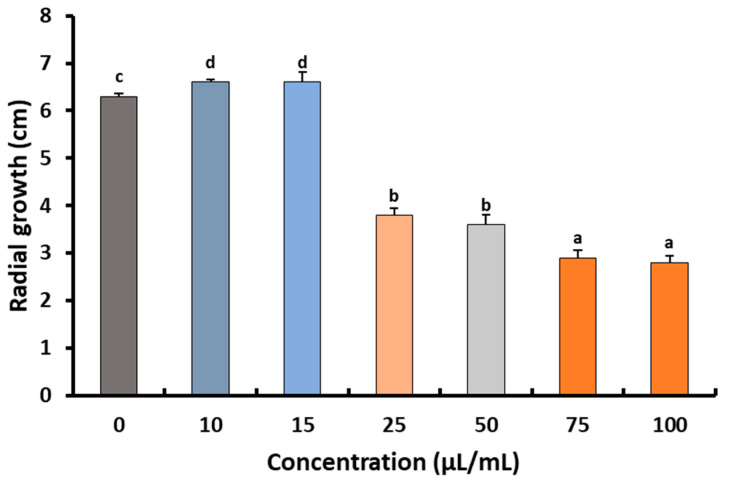
Impacts of different concentrations of preen oil on *B*. *cinerea* radial growth. Vertical bars represent the standard error (*n* = 3). Columns followed by the same letter are not significantly different at *p* < 0.05.

The quantity of extractable preen (uropygial) oil from white chicken feathers was 9.43 mg/g of feathers. The fungicidal effect of different concentrations of extractable preen oil was estimated against *B*. *cinerea* Str5, and revealed a significant reduction (*p* < 0.05) in both radial growth and colony-forming fungal units of the pathogen (Figure 3 and Figure 4). The obtained results indicate that the minimum inhibitory concentration (MIC) of the tested preen oil was 25 μL/mL, which reduced the radial growth of *B*. *cinerea* Str5 by 41.3% compared to the control, and decreased the colony-forming units by 28.6%. On the other hand, when increasing the concentration of preen oil, a significant decrease (*p* < 0.05) in fungal radial growth and the number of colony-forming units was observed (Figure 3 and Figure 4). Accordingly, the concentration of 25 μL/mL of preen oil was used as the minimum inhibitory concentration to reduce the severity of the *Botrytis* grey mold on strawberries in vivo.

### 2.3. Chemical Analysis of the Preen Oil Extract

**Figure 5:**
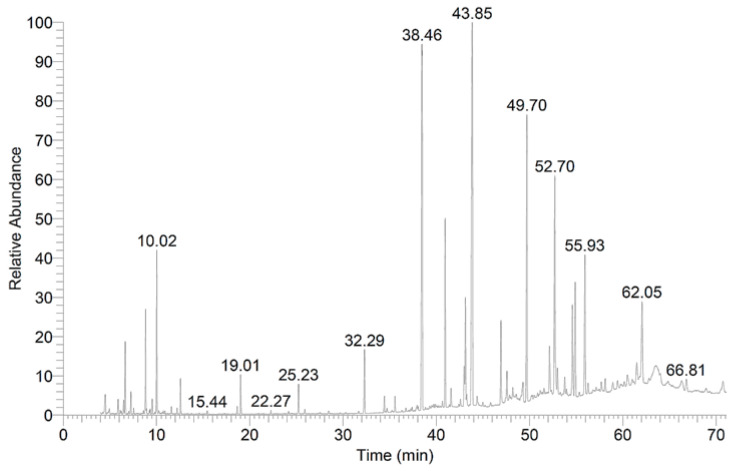
GC/MS profile of preen oil composition.

The analysis of preen oil composition using GC/MS revealed that this oil contains 17 fractions (Figure 5), which were recorded as the main constituents, and accounted for 79.3% of the total obtained analytes (Table 1). The most common components were hexadecenoic acid (14.79%), heptadecanoic acid (4.74%), octadecanoic acid (15.93%), methyl 18-methylnonadecanoate (8.40%), dodecanoic acid (6.01%), and 1,2-benzenedicarboxylic acid (4.34%), whereas 1,3-dimethyl-benzene, octamethyl cyclotetrasiloxane, 1-chloro-4-methyl-benzene, methyl 12-methyl-tridecanoate, 9,12-octadecadienoic acid (Z,Z)-, trans-13-octadecenoic acid, octadecanoic acid-17-methyl-, methyl 14-methyl-eicosanoate, 9,10-dichloro-octadecanoic acid, methyl 20-methyl-heneicosanoate, and methyl 14-methyl-eicosanoate were recorded at 1.78, 1.90, 3.25, 1.75, 1.02, 2.88, 2.27, 1.43, 2.78, 3.35, and 2.69%, respectively.

### 2.4. Screening for Antagonistic Activity of Endophytic Bacteria against B. cinerea Str5 Growth In Vitro

**Figure 6:**
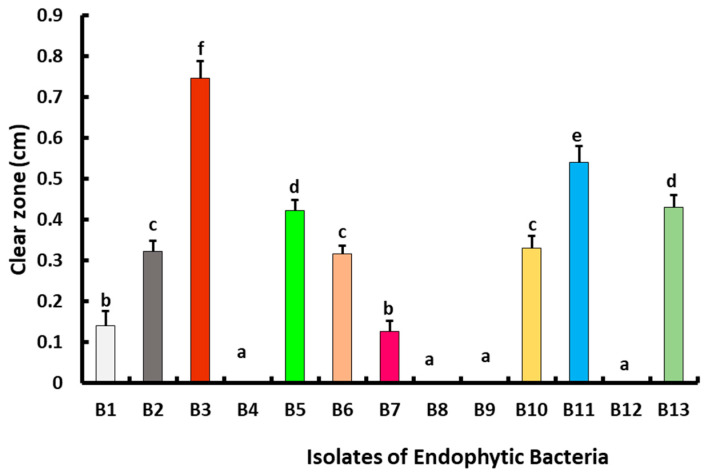
Antagonistic activities of isolated endophytic bacteria against *B*. *cinerea* Str5. Vertical bars represent the standard error (*n* = 3). Columns followed by the same letter are not significantly different at *p* < 0.05.

**Figure 7:**
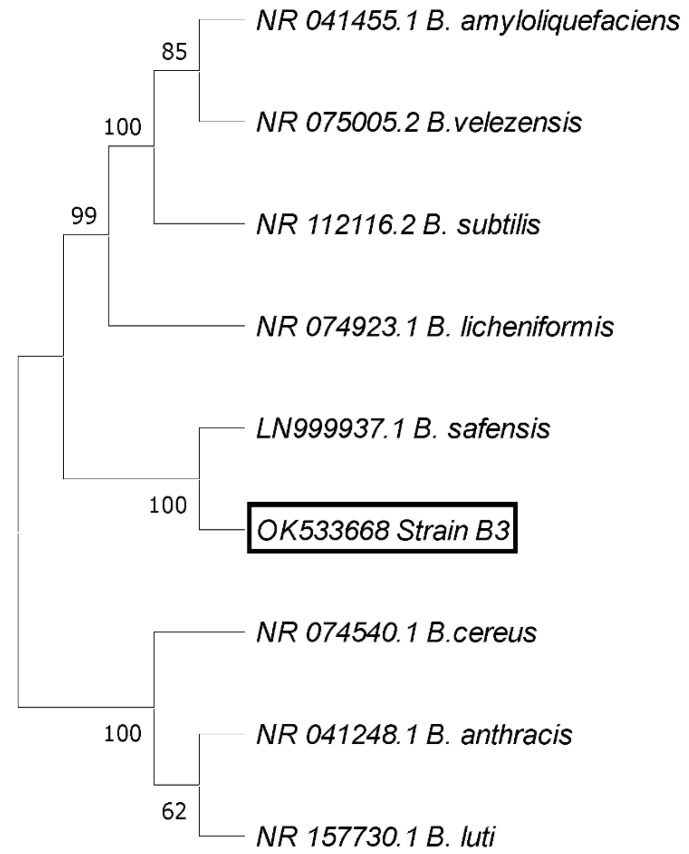
Phylogenetic tree of the most antagonistic bacterial strain B3. The strain is aligned in the clade with *B*. *safensis*, with 100% similarity. The evolutionary history was inferred using the UPGMA method. The evolutionary distances were computed using the maximum composite likelihood method, and are in the units of the number of base substitutions per site.

Out of 13 endophytic bacterial isolates obtained from asymptomatic strawberry fruit, 9 bacterial isolates showed an antagonistic potential against *B*. *cinerea* Str5. The highest antagonistic activity was recorded in the case of isolate B3, giving a clear zone of 0.7 cm, followed by endophytic bacterial isolate B11 (0.5 cm), and then by endophytic bacteria B5 and B13, each recording a clear zone of 0.4 cm (Figure 6). The most prevalent antagonistic bacterial isolate B3 was selected for further in vivo experiments, and was identified based on the morphology of the colony, microscopic, and biochemical characteristics, while the 16S rRNA gene phylogenetic analysis was assessed. The endophytic bacteria displayed positive reactions to fructose, citrate, sorbitol, glycerol, glucose, maltose fermentation, gelatin, casein hydrolysis, and catalase production. On the other hand, the isolated bacteria exhibited negative reactions to galactose fermentation (Table 2). The 16S rRNA partial sequence of 1397 base pairs of antagonistic bacterial isolate B3 showed a 98.73% similarity in *Bacillus safensis* BSKr1 (LN999937). Consequently, based on the phenotypic and genotypic characterization, the antagonistic bacterial strain B3 was identified as *B*. *safensis*, and the obtained sequence was deposited in GenBank under the accession number OK533668. The phylogenetic reconstruction of a 16S rRNA dataset is shown in Figure 7.

### 2.5. Hydrolytic Enzyme Activity of Antagonistic Bacteria

The obtained data in Table 3 show that the endophytic bacterium *B*. *safensis* B3 had the potential to produce hydrolytic enzymes (chitinase, lipase, and protease) that are responsible for the hydrolysis of the *B*. *cinerea* Str5 cell wall, and consequently restrict the fungal growth and spread in the culture medium. The activity of chitinase enzymes produced by *B*. *safensis* was 3.69 ± 0.31 U/mL, and the chitinase-specific activity was 9.66 ± 1.04 U/mg protein. The hydrolytic lipase and protease activities were 10.65 ± 0.51 and 13.28 ± 0.65 U/mL, respectively. The corresponding specific activities of these enzymes were 16.45 ± 3.55 and 18.85 ± 3.26 U/mg protein, respectively (Table 3). 

### 2.6. Efficiency of B. safensis B3 and/or Preen Oil in the Control of Postharvest B. cinerea Str5 Grey Mold on Strawberries, and Consequent Impact on the Fruit

#### 2.6.1. Disease Severity

**Figure 8:**
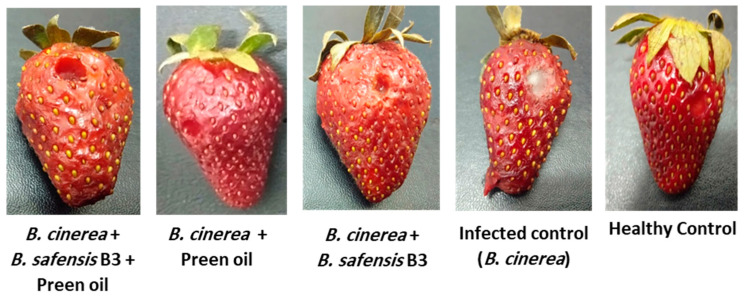
Fruit treatments with preen oil and/or *Bacillus safensis* B3 compared with untreated fruit (healthy control) and fruit infected only with *B*. *cinerea* Str5 (infected control).

The data in Table 4 show that *B*. *cinerea* Str5-infected strawberry fruit recorded a disease severity of 86.11 ± 8.1%, whereas the application of *B*. *safensis* B3 and preen oil—individually or in combination—decreased the disease severity of *B*. *cinerea* Str5 grey mold on strawberry fruits significantly (*p* < 0.05) in vivo (Figure 8). The highest reduction in disease severity was recorded by preen oil (87.25%), followed by the application of *B*. *safensis* (86.52%). However, the combinations of both treatments did not show any stimulation of the biocontrol efficiency, but the reduction in the disease severity was lower than that caused by either of the individual treatments, at 77.28% (Table 4).

#### 2.6.2. Biochemical Analysis

##### Total Phenolics

**Figure 9:**
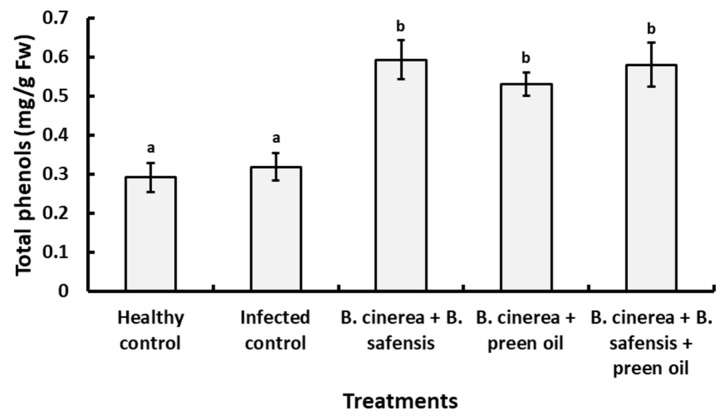
Effects of different treatments on total phenols in strawberry fruit. Vertical bars represent the standard error (*n* = 3). Columns followed by the same letter are not significantly different at *p* < 0.05.

The data in Figure 9 reveal that both *B*. *safensis* and preen oil significantly increased the total phenol contents of infected strawberry fruit when applied either individually or in combination (*p* < 0.05), compared to the healthy or the infected control fruit.

##### Antioxidant Enzymes

**Figure 10:**
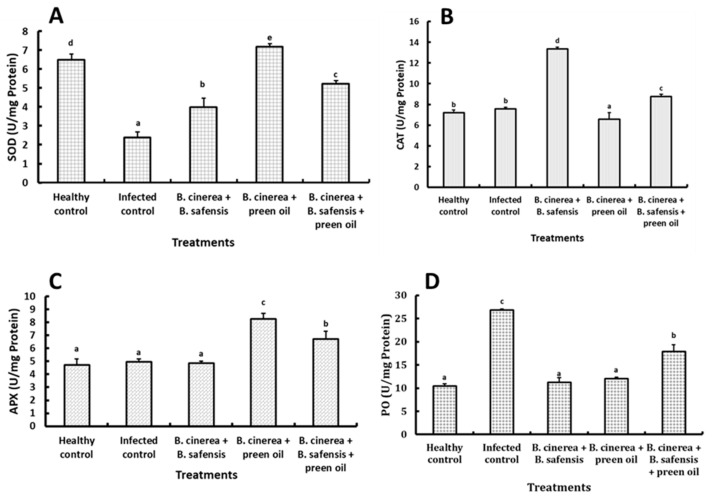
Effects of different treatments on antioxidant activities of superoxide dismutase (SOD) (**A**), catalase (CAT) (**B**), ascorbate peroxidase (APX) (**C**), and peroxidase (PO) (**D**) in strawberry fruit. Vertical bars represent the standard error (*n* = 3). Columns followed by the same letter are not significantly different at *p* < 0.05.

The obtained results display the activity of antioxidant enzymes attributed to the applied bioagents endophytic *B*. *safensis* and preen oil, individually or in combination, in the infected strawberry fruit (Figure 10). The infected strawberry fruit showed a remarkable decrease in superoxide dismutase (SOD) compared with the healthy fruit, whereas the infected fruit inoculated with the bioagents displayed a significant increase (*p* < 0.05) in the SOD activity compared with fruits infected only with *B*. *cinerea* Str5 (Figure 10A). On the other hand, the inoculation of infected strawberry fruit with *B*. *safensis* B3 showed a significant increase (*p* < 0.05) in catalase (CAT) activity compared with other treatments (Figure 10B). Furthermore, the application of the bioagents increased the ascorbate peroxidase (APX) activity significantly (*p* < 0.05) compared with infected strawberry fruit (Figure 10C). Infection of strawberry fruit with *B*. *cinerea* Str5 exhibited a high enzyme activity of peroxidases (PO) compared with healthy fruits (Figure 10D), whereas the application of the bioagents decreased the activity of peroxidases compared with infected strawberry fruit.

## 3. Discussion

In this study, *Botrytis cinerea* Str5 was isolated from strawberry fruit with grey mold disease. The pathogenicity test of *B*. *cinerea* Str5 showed a high disease severity on the strawberry fruit with a high level of fungal colonization. *Botrytis cinerea* is considered to be one of the most problematic necrotrophic pathogens in the fruit sector, especially in the storage stage, as more than 586 plant species can be infected by this necrotrophic fungus [21] resulting in an economic loss of millions of USD. Annually, millions of USD are spent for the control of this fungus via chemical fungicides [22]. Due to economic and environmental concerns, as well as fungicide resistance [23], there is an urgent need for alternative strategies for *B*. *cinerea* control. This study explored the biocontrol efficacy of the novel and eco-friendly bioagents preen oil and endophytic *Bacillus safensis* for controlling postharvest *Botrytis* grey mold in strawberry fruit.

The fungicidal effects of different concentrations of preen oil against *B*. *cinerea* Str5 showed a significant reduction in both radial growth and colony-forming units of the pathogen. Our findings are supported by those of Alt et al. [24], who reported that the preen oil extracted from the uropygial glands of birds acts as an important line of defense against harmful microorganisms. Interestingly, some studies have investigated the applicability of preen oil as an antimicrobial agent and to prevent attack from parasites and pathogens [25,26]. Moreover, Cook et al. [27] reported that purified preen oil can be used as a safe human food and animal feed additive to provide benefits for human and animal health, with many applicable medicinal properties [27]. Recently, there has been great interest in the application of bioactive compounds for controlling fungal pathogens on pre- and postharvest crops and food products, because these bioactive compounds are renewable, safe for humans, and eco-friendly [28]. Although there are many studies concerning the investigation of the fungicidal activity of plant extracts and essential oils against various pathogenic fungi [29], no studies have been performed to assay the bioactivity of preen oil extracted from birds’ feathers in controlling phytopathogenic fungi. Consequently, the current study is considered a pioneer in discovering the biofungicidal effect of preen oil against *B*. *cinerea* Str5 diseases in strawberries. Additionally, the quantity of extractable oil from chicken feathers was 9.34 mg/g of feathers. This could encourage the application of the oil as an innovative strategy to manage postharvest disease in strawberries on a commercial scale.

The GC/MS analysis of preen oil revealed the presence of many fatty acids, lipids, and other hydrocarbon components, e.g., hexadecenoic, heptadecanoic, dodecanoic, and 9,12-octadecadienoic acids, as well as methyl nonadecanoate, methyl eicosanoate, and 1,3-dimethyl-benzene. Interestingly, Lattanzio et al. [30] stated that the component dimethoxybenzoic acid and its derivative compounds exhibited an important role in the suppression of *B. cinerea* postharvest diseases [31]. Additionally, fatty acids of the phospholipids are vital constituents of the two-layered lipids in the cell membrane, which retains the cell structure and integrity [32]. Furthermore, these phospholipids play an important role in controlling mechanisms of plant diseases—either directly, by involving the components in inducing disorders and damages into the plasma membranes, or indirectly, by preventing the biosynthesis pathways of fatty acids that target the plasma membrane phospholipids [32]. Moreover, the polyunsaturated 9,12-octadecadienoic acid (linoleic acid, 18:2) showed antifungal properties against many plants’ pathogenic fungi [33]. Consequently, the chemical analysis of the preen oil obtained from GC/MS revealed that this extracted oil has potential antifungal activity against the *Botrytis* postharvest diseases and, for that reason, preen oil could be applied as a new and innovative strategy for prospective applications in agricultural technologies and food safety.

Furthermore, there is currently great interest in searching for antagonistic strains that have biocontrol potential for postharvest diseases caused by bacteria, yeasts, or fungi [34,35]. Moreover, there is still a great need for new effective microbial species from unexploited environments that could act as biocontrol agents. In the present study, the endophytic bacterium *Bacillus safensis* B3 exhibited the highest antagonistic activities against *B*. *cinerea* Str5; consequently, it was chosen to evaluate the potential to produce fungal cell-wall-hydrolyzing enzymes and their capability to reduce *Botrytis* grey mold severity in vivo. Antagonistic endophytic bacteria are ubiquitous microbial agents that can colonize and inhabit the interior asymptomatic plant tissues without triggering any harmful impacts [36,37], displaying intimate interactions with the host plant; as such, they receive considerable attention as biocontrol agents, and provide protection against postharvest diseases [38,39]. Many authors have revealed that *Bacillus safensis* is considered to be a plant-host-dwelling endophytic bacterium, and showed important sources for innovative applications including recyclable microbial enzymes, drug delivery, synthetic biology, and bioactive metabolites that are crucial for numerous biotechnological applications [40,41]. Remarkably, Wu et al. [41] stated that *B. safensis* isolated from *Chloris virgata* root showed a high potential for hydrocarbon degradation and plant-growth activities. On the other hand, *B. pumilus* and *B. safensis* are considered to be the most widespread and significant soil bacterial species within the *B. pumilus* group [42], and exhibit potent biotechnological applications, such as in probiotics and phytosanitary-based products [43,44]. Lateef et al. [45] stated that *B. safensis* is considered to be a safe industrial candidate due it being harmless to humans, producing no toxic metabolic byproducts, and retaining a potent secretion system for extracellular proteins. Due to these features, *B. safensis* may find feasible applications in medicine, agriculture, and other industries, including probiotics, amino acids, and vitamins. Based on these findings, *B. safensis* could be used as a safe biocontrol agent against postharvest fruit diseases.

Interestingly, the endophytic bacterium *B*. *safensis* B3 had the potential to produce hydrolytic enzymes (chitinase, lipase, and protease) that are responsible for the hydrolysis of the *B*. *cinerea* Str5 cell wall, consequently restricting fungal growth. One of the proposed mechanisms for the biological control of fungal infections and plant pathogen inoculum potential is the degradation of pathogens’ cell walls by hydrolytic enzymes [46,47]. The ability of the endophytic bacterial isolate *B*. *safensis* to produce hydrolytic enzymes revealed its potential as a biocontrol agent against pathogenic fungi. Similar to our results, many biocontrol microorganisms have shown the potential to produce cell-wall-degrading enzymes (chitinase, lipase, protease, and CMCase), such as *Trichoderma* spp. [48], *Wickerhamomyces anomalus* [49], *Talaromyces pinophilus* [50], and *Bacillus subtilis* [47]. *Bacillus safensis* is found in various environments, and shows diverse benefits for plant growth, showing plant-growth-promoting activities, antagonistic activities against fungal growth, and the ability to reduce the growth of phytopathogens [51]. Consequently, the capability of the endophytic bacterium *B*. *safensis* to produce extracellular cell-wall-degrading enzymes is often used as a prevalent indicator for its pathogen-antagonistic characteristics [52,53].

In the present study, *B*. *cinerea* Str5 showed a disease severity of 86.11 ± 8.1% in infected strawberry fruit, whereas the application of the bioagents *B*. *safensis* B3 and preen oil individually decreased the disease severity of *B*. *cinerea* Str5 grey mold on strawberry fruit in vivo by 87.25% and 86.52%, respectively. Meanwhile, the combination of both bioagents did not show any stimulation of the biocontrol efficiency. We assume that the negative impact of the mixture could be because the preen oil has an inhibitory effect on *B*. *safensis* in some ways that could obscure or inhibit the efficiency of the bacteria. The main implication of these results is that in practice, and from the economic point of view, either the preen oil or *B. safensis* may be used separately, while their combination should be avoided.

Furthermore, the in vivo application of either *B. safensis* or preen oil increased the total phenols, superoxide dismutase (SOD), and ascorbate peroxidase (APX) activity in infected fruit compared to the healthy or the infected strawberry fruit. Additionally, the inoculation with preen oil led to a significant decrease in catalase (CAT) enzyme activity in infected strawberry fruit, whereas the application of the preen oil and *B. safensis* decreased the activity of peroxidases. The increase in phenolic contents in strawberry tissues may indicate the strength of plant resistance against *Botrytis* grey mold [54] due to the enhancement of the strawberry cell wall’s mechanical strength [55], in addition to the reductions in water permeability and apoplastic solute conductance that lead to increased cell resistance against pathogens [56]. Osbourn [57] stated that the deposition of phenolics may be toxic to the phytopathogens that act as substrates for oxidative reactions producing toxic quinones [58], as well as increasing the plant resistance due to changing the pH value of the cells’ cytoplasm, and subsequent inhibition of the fungal pathogens [59]. Interestingly, the changes in the antioxidant enzymes’ activity elucidate the role of the bioagents in the enhancement of the plant defense system through detoxification of toxic reactive oxygen species (ROS) that are initiated by the infection process. The infection process with the pathogenic fungi may be associated with prompt peroxidase activity (PO) [60] and, consequently, induced plant defense strategies, including lignin biosynthesis, deposition of plant cell walls, and reactive oxygen species generation [61]. Consequently, the antioxidants APX and CAT may play a pioneering role in scavenging hydrogen peroxides [62].

## 4. Materials and Methods

### 4.1. Plant Materials

Strawberry fruit (*Fragaria ananassa* Duch.) infected with *Botrytis* grey mold were collected from commercial markets in the city of Assiut, Egypt, and Abha city, Saudi Arabia, in sterile bags and transferred to the laboratory for isolation of the fungal pathogen.

### 4.2. Isolation of Fungal Pathogen

The pathogenic fungus (*B. cinerea* Str5) was isolated from the infected strawberries via surface sterilization, as described by Abdel-Hafez et al. [63]. The collected fruit were washed several times with tap water and then rinsed with sterilized water. Small pieces of infected parts, ~1 cm^2^ in size, were surface-sterilized using 70% ethanol for 1 min, 2% sodium hypochlorite for 1 min, and 70% ethanol for 30 s. Finally, the pieces were washed with sterilized H_2_O and air-dried under sterilized conditions [63]. The sterilized fruit pieces were inoculated on the surface of potato dextrose agar (PDA) medium and incubated at 25 ± 2 °C until fungal growth. The growing pathogen was purified on PDA plates and identified based on the determination of its morphological features in terms of single-spore cultures as well as microscopic features of conidia and conidiophores [64], and then it was preserved and maintained on a PDA slant at 4 °C in the culture collections of the mycological herbarium under culture number Str5 in the Botany and Microbiology Department, Faculty of Science, Assiut University.

### 4.3. Assay for Pathogenicity Test of B. cinerea Str5 on Strawberry Fruit

To verify the pathogenicity of the fungal pathogen, the recovered fungal isolate was used as an inoculum to infect healthy strawberry fruit, in order to estimate its ability to cause *Botrytis* grey mold disease. Visually healthy fruit were washed with distilled H_2_O and then sterilized with 70% ethanol for 3 min, followed by washing with sterilized distilled water and air-drying under sterilized conditions. A sterilized cork borer (0.5 cm in diameter) was used to make three wounds on healthy fruit. Then, 50 μL of *B*. *cinerea* Str5 conidial suspension (10^7^ conidia/mL) was inoculated into each wound. Healthy strawberry fruit with wounds exposed to 50 μL of sterile distilled H_2_O were used as controls. The inoculated fruit were incubated at 25 ± 2 °C in sterile plastic boxes containing sterilized wetted cotton for 7 days. For each treatment, 3 fruit were used as replicates. The disease virulence of the tested fungal isolate was identified by the development of *Botrytis* grey mold fungus on infected healthy fruit [65].

### 4.4. Scanning Electron Microscopy Examination

Small pieces from healthy and infected strawberry fruit were fixed in 2% glutaraldehyde solution in 50 mM phosphate buffer solution, pH 7.2, and preserved at room temperature [66]. The samples were investigated using a scanning electron microscope (JEOL JSM-5400 LV, 15 KV) at the Electron Microscopy Unit, Assiut University, Egypt. All of the obtained images were computer-processed.

### 4.5. Preparation and Assay of Biocontrol Agents

#### 4.5.1. Preen Oil

##### Extraction and Fungicidal Activity of Preen Oil

White chicken feathers were collected from slaughterhouses in Assiut, Egypt, and then washed with cold sterile water, before being air-dried for 2 days. For extracting preen oil, 100 g of dried white chicken feathers was added to 200 mL of extracting solvent (chloroform:methanol 2:1) and then shaken at 120 rpm for 24 h. The mixture was filtered using a porcelain funnel at room temperature. The supernatant was centrifuged at 6000 rpm for 10 min in order to remove coarse particles, and then evaporated under vacuum to obtain a volume of 5 mL and, finally, sterilized using a Seitz bacterial filter (0.22 μm). The collected extracts were preserved in sterilized brown vials at 4 °C until being used for assaying their biocontrol potential against *B. cinerea* Str5 [67]. For investigation of the fungicidal activity of preen oil, the disk diffusion technique was used [68]. Sterilized disks (6 mm) were loaded with 100 µL of the extracted preen oil and were placed in 9 cm PDA plates seeded with the fungal pathogen (10^7^ conidia/mL). Sterile n-hexane-loaded disks were used as controls, and then the plates were incubated at 28 ± 1 °C for 7 days. The antagonistic effect of the oil extract was assessed by measuring the diameter of the inhibition zones (mm) as described by Andrews [69]. The assays were performed in triplicate.

##### Effects of Preen Oil on the Fungal Pathogen (Linear Growth and Fungal Colony-Forming Units)

The antagonistic effect of preen oil was estimated in vitro against *B. cinerea* Str5, by mixing 20 mL of potato dextrose agar (PDA) medium with different concentrations of the preen oil (10, 15, 25, 50, 75, and 100 µL/mL). The PDA plates were inoculated with 5 mm diameter *B. cinerea* Str5 agar disks (7-day-old culture). Three plates were used as replicates for each concentration [70], and PDA plates without the preen oil were used as controls. After 7 days of incubation, the reduction percentage of the *B. cinerea* Str5 linear growth was measured as described by Bouziane et al. [71]. For estimation of the fungal colony-forming units grown on each prepared concentration (10, 15, 25, 50, 75, and 100 µL/mL), 1 mL of fungal spores (10^7^ conidia/mL) was spread on the surface of PDA plates (amended with rose bengal) containing preen oil. The inoculated plates were incubated at 25 ± 2°C for 7 days, and then the growing fungal colonies were estimated as colony-forming units (CFU/mL). Three plates were used as replicates for each concentration. Minimum inhibitory concentration (MIC) was estimated as the lowest concentration of preen oil that inhibited the pathogen growth on the agar plates [19].

##### Analysis of Preen Oil Composition

The chemical composition of preen oil extracted from white chicken feathers was analyzed by using GC/MS (Thermo Fisher Scientific, Model: DPC-Direct Probe Controller (DPC- 20451), Waltham, MA, USA; at the Chemistry Department, Faculty of Science, Assiut University), as described by Najjar et al. [72].

#### 4.5.2. Antagonistic Bacteria

##### Isolation of the Endophytic Bacteria

Endophytic bacterial isolates were recovered from fresh healthy strawberry fruit via surface sterilization. Healthy fruit were sterilized using 70% ethanol for 1 min, followed by 2% sodium hypochlorite for 1 min. Detached segments from the healthy fruit were washed with sterilized distilled water, air-dried under aseptic conditions, and then inoculated on the surface of nutrient agar plates, after which they were incubated at 28 °C for 48 h. The growing bacterial colonies from the margins of fruit segments were purified using the streak plate technique for single colonies [73], and then maintained on a nutrient agar slant at 4 °C in the culture collections of the Botany and Microbiology Department, Faculty of Science, Assiut University.

##### Assay for Antagonistic Activity

The antagonistic activity of 13 endophytic bacterial isolates (isolated from healthy strawberry fruit) against *B*. *cinerea* Str5 was assayed as described by Hashem and Alamri [65], with some modifications. The endophytic bacterial isolates were grown on nutrient agar plates seeded with *B*. *cinerea* Str5 inoculum (10^7^ conidia/mL), and then incubated at 28 ± 1 °C for 7 days. Three plates were used as replicates for each isolate. Finally, the fungal growth inhibition was assessed by the determination of the inhibition zone (mm), as measured from the edge of the fungal growth to the edge of the bacterial growth. Three replicates were used for the assay.

##### Identification of the Most Antagonistic Endophytic Bacterial Isolate

The most prevalent antagonistic bacterial isolate was identified based on culture, microscopic, and biochemical tests, as described in *Bergey’s Manual of Systematic Bacteriology* [74]. The identification of antagonistic bacteria was molecularly confirmed by amplification of the partial 16S rRNA gene, using the primers 27F and 1492R [75]. The PCR amplification was achieved in a reaction volume (25 μL) containing template DNA (10–50 ng), 0.4 μM of each primer, 0.75 U of EF-Taq DNA polymerase (SolGent, Daejeon, Korea), 0.2 mM of each d NTP (SolGent, Daejeon, Korea), and 1× EF-Taq reaction buffer (SolGent, Daejeon, Korea). The thermocycling conditions involved an initial denaturation step at 95 °C for 15 min, followed by 30 cycles at 95 °C for 20 s, 50° C for 40 s, and 72° C for 1.5 min, with a final extension step at 72° C for 5 min. The PCR product was separated by gel electrophoresis on 1.5% agarose containing ethidium bromide with a 0.5× Tris-acetate-EDTA (TAE) buffer, and visualized using a UV illuminator. The PCR product was then purified using a SolGent PCR purification kit (SolGent, Daejeon, Korea), according to the manufacturer’s instructions [76]. The amplified 16S rRNA gene was sequenced using an ABI BigDye Terminator (v 3.1) cycle sequencing kit (Applied Biosystems, Foster City, CA, USA) and an ABI 373 0XL DNA analyzer (Applied Biosystems, Foster City, CA, USA). The obtained sequence was blasted against the NCBI GenBank database (https://blast.ncbi.nlm.nih.gov/Blast.cgi) accessed on 10 October 2021, using the BLAST search program to evaluate the degree of DNA similarity.

##### Assay of Hydrolytic Activity of the Most Antagonistic Bacteria

- Determination of chitinase activity

The chitinase activity of the most antagonistic bacterial isolate was estimated by growing the bacterial isolate in a medium [50] containing colloidal chitin, 1 g L^−1^; (NH_4_)_2_SO_4_, 1.4 g L^−1^; NaH_2_PO_4_, 6.9 g L^−1^; KH_2_PO_4_, 2.0 g L^−1^; peptone, 10 g L^−1^; and MgSO_4_.7H_2_O, 0.3 g L^−1^ at 28 °C for 48 h. The reaction mixture was assayed by mixing 1 mL of colloidal chitin in 0.05 M Tris-HCl buffer, pH 5.5, and 1 mL of bacterial filtrate, and then incubated at 30 °C for 1 h. At the end of the incubation, 1 mL of 3,5-dinitrosalicylic reagent was added to the reaction mixture, and the chitinase activity was assessed by determining the increase in reducing groups [77] via spectrophotometric methods at 540 nm, compared against a control tube (dist. H_2_O). The assay was repeated thrice (*n* = 3). Chitinase enzyme units were determined as the amount of chitinase enzymes required to liberate 1 µM of reducing groups (N-acetylglucosamine) per min [78].

b. Determination of protease activity

The protease enzyme activity of the antagonistic bacterial isolate was assayed by growing it on a sterilized broth medium containing tryptone, 10.0 g L^−1^; peptone, 5.0 g L^−1^; (NH_4_)_2_SO_4_, 3.0 g L^−1^; K_2_HPO_4_, 2.0 g L^−1^; MgSO_4_ 0.2 g L^−1^; and casein, 1.0 g L^−1^ [79], and incubated at 28 °C for 72 h under shaking (120 rpm). The reaction mixture was assayed by mixing 5 mL of casein solution (1%) and 1 mL of bacterial filtrate by vortexing, followed by incubation in a water bath at 30 °C for 10 min. After that, 5 mL of trichloroacetic acid (10%) was added to the reaction mixture to stop the enzymatic reaction, and then 5 mL of Na_2_CO_3_ and 1 mL of Folin–Ciocalteu reagent were added. Protease enzyme activity was assessed by the determination of the amount of liberated amino acids, by measuring the absorbance spectrophotometrically at 660 nm and using tyrosine as the standard curve. The assay was repeated thrice (*n* = 3). Protease activity units were estimated as the amount of protease enzymes required to release 1 μ mol tyrosine per min.

c. Determination of lipase activity

Lipase enzyme activity was estimated by the cultivation of antagonistic bacterial isolate on Tween-containing medium, including K_2_HPO_4_, 1.5 g L^−1^; NH_4_NO_3_, 1 g L^−1^; MgSO_4_, 0.025 g L^−1^; CaCl_2_, 0.025 g L^−1^; FeSO_4_, 0.015 g L^−1^; and ZnSO_4_, 0.005 g L^−1^. The pH was adjusted to 5.0 and the medium was supplemented with 10 mL L^−1^ Tween 80 [80]. The cultures were incubated at 28 °C and 120 rpm for 7 days. Lipase enzyme activity was assayed with p-nitrophenylpalmitate (pNPP), as described by Prazeres et al. [81], using a spectrophotometric method at 410 nm. Lipase activity units (U/mL) were calculated as the amount of lipases required for the production of 1 μ mol nitrophenol per min. The assay was repeated thrice (*n* = 3). Enzyme-specific activity was calculated as enzyme units per mg protein, and protein concentrations of the culture were assayed as described by Lowry et al. [82].

### 4.6. Biocontrol Efficiency of B. safensis B3 and Preen Oil against Postharvest Botrytis Grey Mold on Strawberries In Vivo

Healthy strawberry fruit were selected and surface-sterilized with 70% ethyl alcohol and 2% NaOCl solution. Then, the fruit were washed with sterile distilled water and left for drying under sterilized conditions. A sterilized cork borer was used to make an equal number of wounds (two wounds) on each strawberry fruit [83]. The wounded fruit were then inoculated with a spore suspension of *B. cinerea* Str5 (10^7^ spore/mL), along with the bioagents according to the following treatments: (1) strawberry fruit inoculated with 25 µL of fungal spore suspension (infected control), (2) strawberry fruit inoculated with 25 µL of sterilized n-hexane (healthy control), (3) strawberry fruit inoculated with 25 µL of *B. cinerea* Str5 spore suspension and 25 µL of preen oil, (4) strawberry fruit inoculated with 25 µL of *B. cinerea* Str5 spore suspension and 25 µL of antagonistic bacteria (10^5^ CFU/mL), and (5) strawberry fruit inoculated with 25 µL of *B. cinerea* Str5 spore suspension and 25 µL of preen oil + 25 µL of antagonistic bacteria (10^5^ CFU/mL). Three fruit were used as replicates for each treatment. After that, inoculated fruit were left under sterilized conditions for drying for 1 h. Then, strawberry fruits were incubated at 25 ± 2 °C for 7 days in a plastic box, and the relative humidity was maintained at 98%.

#### 4.6.1. Assay of Disease Severity

The inoculated strawberry fruit were examined to detect *B. cinerea* colonization and weighed. Then, the decayed tissues were collected, the remaining fruit parts were weighed, and the disease severity was assessed as described by Yaganza et al. [84].

Disease severity index (DSI) was determined by the following equation:

DSI = (A − B/A) × 100

where A is the strawberry fruit weight with rotting, and B is the strawberry fruit weight without rotting.

#### 4.6.2. Biochemical Analysis of the Inoculated Strawberry Fruit

The biochemical variations were estimated in the inoculated strawberry tissue of the previous treatments.

- Total phenol content

Strawberry fruit segments were dipped in liquid N_2_ and homogenized with 80% methanol (0.1% *w*/*v*). The fruit homogenate was centrifuged at 10,000 rpm at 4 °C for 15 min, and the supernatant was evaporated at 65 °C with a rotary evaporator. The residual parts were dissolved in 80% methanol (5 mL). Three replicates were used for each treatment. The total phenolic content of the strawberry samples was assessed using Folin–Ciocalteu reagent, as described by Kofalvi and Nassuth, [85], and the absorbance was determined at 750 nm. The assay was repeated thrice (*n* = 3). The total phenolic contents were expressed as mg/g FW [86].

b. Antioxidant enzymes

One gram of strawberry fruit tissues of each treatment was milled in 10 mL of phosphate buffer (100 Mm, pH 6.8) using a mortar and pestle. The resultant homogenates were centrifuged for 5 min at 10,000 rpm under cooling to remove solid residues. The obtained extract was used to determine the antioxidant enzymes. The supernatant of strawberry fruit extract was assayed for estimation of superoxide dismutase (SOD) [87], ascorbate peroxidase (APX) [88], and catalase (CAT) [89]. Peroxidase activity (PO) of strawberry samples was assayed spectrophotometrically at 436 nm as described by Abo-Elyousr et al. [90]. Peroxidase enzyme activity was assayed via changes in absorbance, and expressed as peroxidase-specific activity (OD 436 nm/mg protein). The protein concentration of the strawberry extract was assayed as described by Lowry et al. [82], and bovine serum albumin (BSA) was used as a standard protein. The assay was repeated thrice (*n* = 3).

### 4.7. Statistical Analysis

The quantitative data were statistically analyzed using SPSS 22.0 (SPSS, 2013). All treatments were laid out in 3 replicates (*n* = 3). The data were initially examined for a normal distribution of errors using a Shapiro–Wilk W-test, and for the homogeneity of variance using Levene’s test. Data were analyzed for the significance of variation using a one-way analysis of variance (ANOVA). Means were compared using Tukey’s range test at *p* < 0.05 [91].

## 5. Conclusions

This study confirmed the efficiency of preen oil as well as the endophytic *B. safensis* B3 in the control of *Botrytis* grey mold of postharvest strawberry rot caused by *B. cinerea* Str5. Our results can be considered to be a pioneering report on the application of preen oil in the biological control of plant diseases. The results are very promising, and encourage the application of preen oil in the management of postharvest fruit rot, instead of using microorganisms that could give the fruit an unacceptable appearance for consumers. We recommend the application of preen oil as a new and innovative strategy to keep fruit free from infection after harvesting and during large-scale commercialization. Furthermore, we recommend a test of the efficacy of preen oil on a large number of fruit crops in the future, as well as its formulation into an appropriate biofungicide.

## Figures and Tables

**Table 1:** The components of preen oils extracted from chicken feathers.

RT	The Components	% of Total Constituents
6.65	Benzene, 1,3-dimethyl-	1.78
8.83	Cyclotetrasiloxane, octamethyl-	1.90
10.02	Benzene, 1-chloro-4-methyl-	3.25
32.29	Methyl 12-methyl-tridecanoate	1.75
38.46	Hexadecanoic acid	14.79
40.95	Heptadecanoic acid	4.74
42.99	9,12-Octadecadienoic acid (Z,Z)-	1.02
43.12	trans-13-Octadecenoic acid	2.88
43.86	Octadecanoic acid	15.93
46.93	Octadecanoic acid, 17-methyl-	2.27
49.70	Methyl 18-methylnonadecanoate	8.40
52.13	Methyl 14-methyl-eicosanoate	1.43
52.70	Dodecanoic acid	6.01
54.60	Octadecanoic acid, 9,10-dichloro-	2.78
54.89	Methyl 20-methyl-heneicosanoate	3.35
55.93	1,2-Benzenedicarboxylic acid	4.34
62.05	Methyl 14-methyl-eicosanoate	2.69

**Table 2:** Colonial and biochemical characteristics of the most antagonistic bacterium *B. safensis* B3.

Biochemical Test	Value
Colonial characteristics	
Gram staining	+ *
Colony color	White
Colony shape	Round with irregular margins
Cell shape	Rod-shaped
Biochemical characteristics	
Carbon source utilization	
D-Fructose	+
Citrate	+
D-Sorbitol	+
D-Galactose	−
Glycerol	+
Glucose	+
Maltose	+
Catalase test	+
Gelatin hydrolysis	+
Casein hydrolysis	+
Growth at 4 °C	–
Growth at 37 °C	+
Growth at 45 °C	+

* +: positive reaction; –: negative reaction.

**Table 3:** Enzymatic activities of the endophytic bacterium *B. safensis* B3.

Enzyme	Enzyme Activity (U/mL)	Protein Concentration (mg/mL)	Enzyme-Specific Activity (U/mg Protein)
Chitinase	3.69 ± 0.31 *	0.340 ± 0.29	9.66 ± 1.04
Protease	13.28 ± 0.65	0.705 ± 0.20	18.85 ± 3.26
Lipase	10.65 ± 0.51	0.650 ± 0.14	16.45 ± 3.55

* Value represents the standard error (*n* = 3).

**Table 4:** Impact of *B. Subtilis* and preen oil on postharvest *Botrytis* grey mold severity.

Treatment	Disease Severity (%)
Healthy control	0.00 ± 0.0 *^,a^
Infected control	86.11 ± 0.80 ^c^
*Botrytis* + endophytic bacteria	13.48 ± 0.92 ^b^
*Botrytis* + preen oil	12.57 ± 1.74 ^b^
*Botrytis* + preen oil + endophytic bacteria	22.72 ± 5.13 ^d^

* Value represents the standard error (*n* = 3). Means followed by the same letter are not significantly different at *p* < 0.05.

## Data Availability

The following information was supplied regarding data availability: the sequencing data of the antagonistic bacterial strain *Bacillus safensis* strain B3 were deposited in the NCBI (http://www.ncbi.nlm.nih.gov), accessed on 10 October 2021 website under the accession numbers OK533668.
